# Readability of online health information pertaining to migraine and headache in the UK

**DOI:** 10.1177/20494637221134461

**Published:** 2022-10-18

**Authors:** Kate Atherton, Mark J. Forshaw, Tara M. Kidd

**Affiliations:** 1 School of Psychology, 4589 Liverpool John Moores University, UK

**Keywords:** Headache, migraine, online health information, search engines, self-management, self-care, readability

## Abstract

An estimated 46% of the worldwide adult population live with an active headache disorder, and it is thought that there is a proportion of headache and migraine sufferers who do not attend for medical care, instead choosing to manage their symptoms at home. The internet continues to act as a source of online health information for self-management, however, it is important that this information can be understood by the user. Research indicates that most health information online is written at a level too difficult for much of the UK population to understand. The aim of this study was to investigate the readability of online health information pertaining to headache and migraine for a UK-based internet user accessing the top four search engines. Searches for ‘headache’ and ‘migraine’ were performed on each search engine and results from the first page were selected for analysis. Five validated readability tests were used to analyse readability; Flesch-Kincaid Grade Level, Flesch Reading Ease, Gunning Fog Index, Coleman-Liau Index and Simple Measure of Gobbledygook Index. We found that the majority of online health information about migraine and headache is too difficult for the UK adult population to read. Findings highlight work is required to ensure that information from a wider variety of sources is easier to comprehend for much of the population in order for individuals to make informed decisions about health seeking and self-management of headache and migraine. Health information providers should weave readability analysis into their content design process, incorporating shorter sentences and simpler words in their description of conditions and treatment.

## Introduction

It is estimated that worldwide up to three quarters of adults have had headache in the last year,^1^ and 46% of the adult population are thought to live with an active headache disorder, for which the debilitating symptoms are estimated to be a major cause of disability and economi burden worldwide.^2,3^ Of headache disorders, tension-type headache and migraine are most common^4^; in the UK, migraine is thought to affect around 10 million adults^5^ and accounts for around 2.5 million consultations in primary care every year.^6^ Migraine sufferers commonly report intrusive pain impacting on their ability to carry out daily activities, including work,^7^ as well as nausea, vomiting, visual disturbances and light and sound sensitivity.^8,9^ Despite this, it is often misdiagnosed or underdiagnosed and untreated.^10,11^ In the literature, there is a difference in incidence rates between reporting of migraine for questionnaire-based studies versus those using medical records, which suggests that there is a proportion of headache and migraine sufferers who do not attend for medical care.^12^ This, alongside the suggestion of underdiagnosis, suggests that there could be a large proportion of migraineurs who choose to manage their symptoms at home without prescribed medication.

The internet continues to be an important source of health information, and the amount of content available to consumers is growing every day. Many people seek this information online to self-treat symptoms before seeking diagnosis from a health professional^13,14^ and its access increases opportunity to improve knowledge and understanding of a person’s medical condition and self-efficacy,^13^ enabling them to be more active in managing their condition at home. Holding this knowledge can also decrease uncertainty and worry and improve health decision-making^15^ and confidence to ask more informed questions of health professionals,^16^ thus empowering people to actively engage in their own treatment. In *The NHS Long Term Plan*,^17^ authors draw attention to ‘shared responsibility,’ emphasising the importance in supporting individuals to self-manage their conditions and make informed decisions through the provision of knowledge and information.^18^ It is therefore all the more important that the health information visible to internet users in the UK enables them to do this.

There are a number of factors pertaining to how an individual may interact with online health information. For individuals seeking information about specific health-related issues, although information that suits their needs and motivation may be their primary concerns,^16^ literature suggests that instrumental factors, including quality, trustworthiness and usefulness of information are more important in determining online health seeking behaviour than are psychological factors.^19^

Although the availability of online health information is rising with the continued growth of the internet, some suggest that it could increase inequalities in its access; those who are older, have received less education, have lower socioeconomic status and lower internet skill are less likely to use the internet to seek online information.^20,21^ One explanation for the lack of access for some populations could be that the information presented to them is difficult to appraise; of those accessing health information, online lower e-health literacy has been linked to concerns about incorrect interpretation of information and a feeling of information overload, leading to a reduced self-confidence to accurately judge the information, and a lack of trust in the source and their own ability to interpret the content.^22^

Around 40% of adults in the UK struggle to comprehend and make use of health information targeted at the general population.^23^ This lack of accessibility creates a ‘digital divide’^24^; low health literacy has been linked to lower engagement with preventative health behaviours, a decreased likelihood to access healthcare services appropriately and poorer health outcomes.^25,26^ It is therefore important that publishers of online health information ensure that it accounts for differences in health literacy to increase its accessibility. One way to address this is to improve readability. However, readability analyses of online health information consistently find that that it is in fact unsuitable for a large proportion of the population.^27–30^

There is little literature investigating accessibility, including readability, of online health information pertaining to headache and migraine, and though a recent paper found that headache and migraine focused websites failed readability analyses,^31^ it utilised Google International to identify sources, meaning that search results were not reflective of a UK user’s specific experience. This study therefore used UK-based search engines to understand the readability of headache and migraine information available for the UK population.

## Methods

### Search Strategy

Data on the market share held by leading UK search engines suggest that Google (86.31%), Bing (9.61%) Yahoo (2.36%) and DuckDuckGo (1.01%) are most accessed,^32^ thus this analysis used results from these four. To avoid the interaction of algorithms, and bias caused by location, browsers were accessed in incognito mode and cookies and the cache was cleared prior to each search.

The search, which included the pre-planned key terms ‘migraine’ and ‘headache’, was performed by the first author on 28th November 2021 in Liverpool, UK. The search was limited to the first page of results for each search engine as research suggests that sites on the first page receive 92% of all traffic resulting from an average search.^33^

Duplicates, websites not in English, websites not including information on headache or migraine aimed at adults, websites aimed at clinicians, information behind a paywall, discussion boards, advertisements and newspaper articles were excluded from the analysis. If the search result was a website home page, relevant information pages were analysed and an average score was calculated for the entry. If a page contained an article that was split into different pages (e.g. symptoms, causes and diagnosis), all pages were analysed and an average score was calculated. This did not include linked pages which led to separate information sources from which the original menu items or the landing page could not be easily navigated to. Following primary analysis, pages were grouped into ‘parent’ websites to understand readability of content produced by individual organisations, and a secondary analysis was run.

### Readability analysis

Text was copied into Microsoft Word and all figures, captions, links, advertisements, references and disclaimers were removed. Text was then pasted into an online readability tool, *Readable,* for analysis. Five validated tests were identified for the analysis; Flesch-Kincaid Grade Level (FKGL), Flesch Reading Ease (FRE), Gunning Fog Index (GFI), Coleman-Liau Index (CLI) and Simple Measure of Gobbledygook index (SMOG). Tests use different formulae to calculate readability of a piece of text, thus the combination of scores was identified as a way in which to ensure that more facets of readability were analysed. Where FRE, FKGL and GFI incorporate word and sentence length and syllable count into their formulae, GFI calculates readability using word and sentence length alone. SMOG uses the number of words with three or more syllables in three ten-sentence samples to calculate readability. All tests but FRE result in a score that corroborate with the approximate US grade level required to comprehend the text (i.e. a score of 6 aligns with a 6th grade reading level). FRE score uses a scale from 0 to 100, where a lower score indicates a more difficult readability level (0–30 is very difficult, 30–50 is difficult, 50–60 is fairly difficult, 60–70 is standard, 70–80 is fairly easy, 80–90 is easy and 90–100 is very easy).^34^

Adult literacy levels vary across the four UK nations, where between 1 in 4 and 1 in 8 adults have very poor literacy skills.^35^ Guidance from NHS Health Education England and the Office for National Statistics suggests that health information should be aimed at an average 11 year old, which translates to sixth-grade.^36,37^ This analysis therefore used this recommendation as a basis for whether health information was suitable for the population. An indication of a suitable score for FRE was set at 80–90.

## Results

**Figure 1.**
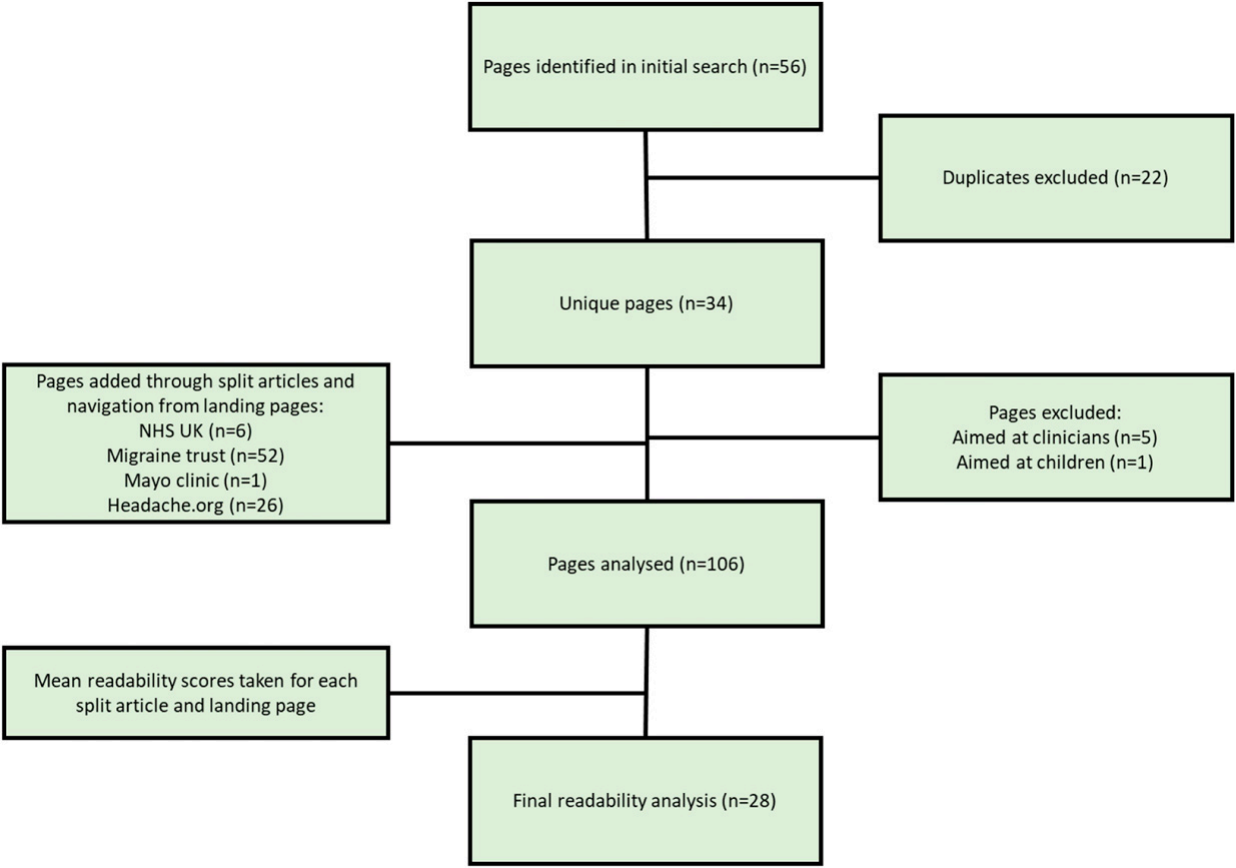
Process for page inclusion and exclusion.

Statistical analyses were performed using SPSS v26. A total of 106 pages were included in the final analysis. Following calculation of means for those linked to home pages and split articles, this resulted in 28 final entries from 17% pages (see Figure 1.) This included 12 for search term ‘migraine’ and 16 for search term ‘headache.’

All five tests indicated that health information on most websites was difficult to read for the majority of the population. Mean grade score (FKGR, GFI, CLI, SMOG) across all websites was 10.01 (SD = 1.70). Mean FRE score was 53.23 (SD = 11.58). This corresponds to an approximate reading age of 15–17 years old, with 7.14% of articles readable for 11–12 year olds. Mean scores for each readability formula are presented in Table 1.

**Table 1.** Readability scores presented by formula.

Readability formula	Mean score	Minimum score	Maximum score	Standard deviation
Flesch Reading Ease	55.23	30.10	78.2	11.58
Flesch-Kincaid grade level	8.19	4.40	11.60	1.82
Gunning Fogg index	9.82	6.90	13.60	1.77
Coleman-Liau index	11.49	6.30	16.90	2.18
Simple Measure of Gobbledygook index	10.55	8.10	13.30	1.38
FKGR, GFI, CLI, SMOG combined score	10.01	6.38	13.20	1.71

Of the pages analysed, the easiest page to read was ‘Headaches’ from NHS UK (M = 6.38, SD = 1.63. According to FRE scores, ‘Headache’ provided by NHS 111 Wales, (78.2) was slightly easier to read than the NHS UK article (77.5). The page most difficult to read was ‘Migraine’ provided by *Wikipedia*, with a mean average grade score of 13.2 (SD =1.15) and an FRE score of 35.7. According to FRE score alone, ‘The complete headache chart’ from *The National Headache Foundation* was most difficult to read (30.10) This scored a mean average grade level of 11.00 (SD = 2.78). See Table 2 for all pages.

**Table 2.** Primary analysis.

Web page title	Organisation	Number of pages analysed	Mean readability score	FRE score
Headaches	NHS UK	1	6.37	77.5
Headache	NHS 111 Wales	1	6.58	78.2
Headache basics	Webmd	1	7.83	71.9
Headaches	NHS Inform	1	8.08	64.6
What is migraine?	Webmd	1	8.40	64.3
Headaches	Cleveland clinic	1	9.10	58.9
Migraine headaches	Cleveland clinic	1	9.13	60.1
Headache	Johns Hopkins Medicine	1	9.13	59.1
Migraine	Patient	1	9.15	61.7
What is causing this headache?	Medical News Today	1	9.33	56.8
Everything you want to know about migraine	Healthline	1	9.60	55.9
Headache: when to worry, what to do	Harvard Health Publishing	1	9.78	60
What is migraine?	Migraine Trust	1	9.80	60
What is migraine?	National Migraine Centre	1	9.98	60.7
14 types of headaches and how to treat them	Healthline	1	10.03	52.1
Headache	Patient	1	10.13	57.8
Everything you need to know about headaches	Healthline	1	10.35	53.2
Migraine	Mayo clinic	2	10.38	52.1
Migraine	NHS UK	7	10.52	53.96
Everything you need to know about migraine	Medical News Today	1	10.63	48.5
Website home page	Migraine Trust	30	10.63	56.74
Types of migraine	Migraine Trust	17	10.98	52.36
Headache - causes	Mayo clinic	1	11.50	36.1
Headache: Types and location	Medicine net	1	11.90	46
Website home page	Headache Management System	27	12.19	44.33
Headache	Wikipedia	1	12.80	37.8
The complete headache chart	National Headache Foundation	1	12.90	30.1
Migraine	Wikipedia	1	13.2	35.7

*Note: ‘Number of pages analysed’ includes instances of articles split over multiple pages and if the search result led to a website landing page.*

Secondary analysis revealed that NHS 111 Wales provided the easiest content to read with a mean grade score of 6.58 (SD = 1.63) and an FRE score of 77.50, indicating an approximate required reading age of 11–13 years old. The highest mean grade score was *Wikipedia* (M = 13.00, SD = 1.26). According to FRE the most difficult content was provided by *The National Headache Foundation* (30.10). *Wikipedia* was second most difficult to read, with a score of 36.75. This indicates an approximate reading age of 17–21 years. See Table 3 for all parent websites.

**Table 3.** Secondary analysis.

Parent organisation	Website URL	Number of search results	Number of pages analysed	Mean readability score	FRE score
NHS 111 Wales	111.wales.nhs.uk/	1	1	6.58	78.2
NHS Inform	Nhsinform.scot	1	1	8.08	64.6
Webmd	Webmd.com	2	2	8.11	68.1
NHS UK	Nhs.uk	2	8	8.45	65.73
Cleveland clinic	my.clevelandclinic.org	2	2	9.11	59.75
Johns Hopkins Medicine	Hopkinsmedicine.org	1	1	9.13	59.1
Patient	Patient.info	2	2	9.64	59.75
Harvard Health Publishing	health.harvard.edu	1	1	9.78	60
Medical News Today	Medicalnewstoday.com	2	2	9.98	52.65
National Migraine Centre	Nationalmigrainecentre.org	1	1	9.98	60.7
Healthline	Healthline.com	3	3	9.99	53.73
Migraine Trust	Migrainetrust.org	3	48	10.47	56.36
Mayo clinic	Mayoclinic.org	2	4	10.94	44.1
Medicine Net	Medicinenet.com	1	1	11.9	46
Headache Management System	Headache.org.uk	1	27	12.19	44.33
National Headache Foundation	Headaches.org	1	1	12.9	30.1
Wikipedia	En.wikipedia.org	2	2	13	36.75

*Note*: ‘Number of pages analysed’ includes instances of articles split over multiple pages and if the search result led to a website landing page.

## Discussion

We found that the majority of online information about migraine and headache is too difficult for the UK adult population to read. Only two pages from the 28 identified were readable at 6th grade level (11–12 year olds). Results from this study are consistent with previous readability analyses of headache and migraine information aimed at an international audience,^31^ and of online health information generally.^38-40^

Our results indicate that articles written and provided by NHS services were amongst the easiest to understand, making up three of the top four scoring organisations in our secondary analysis. Previous research on UK participants found that NHS UK (then NHS Direct) was the most visited website for accessing health information,^41^ thus it is important that its content it provides is easily understood by the population; further, this could improve appropriate access to its own services. In recent years, NHS services have worked to improve readability of online information, providing guidelines and toolkits to content creators, aiming for a reading age of 9–11 years old.^42^ Though some pages analysed did achieve this target, information on migraine was considerably more difficult to understand, being delivered at a grade 10 level (15–16 years old).

Despite the increased access of NHS-owned websites, with presentation of NHS content at the top of all search engine results accessed in this study, it is important to note some other findings. People like to visit multiple websites to get a richer understanding of their condition and to assure the validity of the information found.^43^ Further, the general public may be more likely to access 'dot com' websites over governmental websites when health information seeking^44^ and those with lower health literacy show lower levels of trust in government-owned websites.^45^ It is therefore important to that readable information is available from a wide variety of online sources to ensure that more of the population are able to make informed decisions.

Health information presented by charities can be preferred over government sites,^46^ and of websites analysed as part of this study, there were three charitable providers: *National Migraine Centre*, *Migraine Trust* and *National Headache Foundation*. However, information on these sites was too difficult to understand for much of the UK population, with *National Headache Foundation* content delivered at a level only understandable for those who have accessed higher education. Current UK data suggest that only 42% of the population aged 21–65 are graduates,^47^ meaning potentially important access points to quality information are not able to reach most of the population.

The least readable source of information identified in this study was *Wikipedia*, which stands in line with existing literature.^24^*Wikipedia* has been identified as a prominent source of health information ^48^, and it often appears on the first page of search results. This indicates that health information seekers may access difficult-to-understand information very early on in their searching process.

### Limitations

There are some mostly unavoidable limitations to be considered in interpretation of these findings. Firstly, results were drawn from a cross-sectional analysis at one point in time. It is acknowledged that web pages are updated, and that search engines adapt in response to website traffic and also the previous search behaviours of the individual user.^49^ Further, though the searches conducted did not encompass a time-sensitive topic, a suggestion for future readability analyses would be to undertake the search at different time points to understand whether results change generally. Results nevertheless demonstrate the availability of health information provided by large organisations with high website traffic. Another limitation lies with the search terms used. Authors used ‘headache’ and ‘migraine’ as search terms, but it is acknowledged that some internet users may use phrases or specific symptoms when conducting internet searches.^50^ Although ‘headache’ is a symptom in itself, in the case of migraine, individuals may not understand at the point of searching that this is what they are experiencing, and may try to encapsulate symptoms in a different way, and may be unsure of what keywords to use.^43^ It is also acknowledged that by the nature of headache and migraine being medical conditions, there are instances in which the use of medical terminology is unavoidable, such as on pages denoting treatment options, including sharing the names of available medications. This may mean that there are pages which could have increased readability scores due to the number of syllables in such words. However, authors used a number of different readability analysis tools which used a mix of sentence length, word length and syllables to reach a final score, of which an average was taken.

### Implications and suggestions for future research

This study adds to the existing evidence base by providing an insight into the accessibility of online health information for individuals seeking an understanding of headache and migraine symptoms in the UK, and results increase awareness of the accessibility of information central to self-management and potential patient activation, imperative to the achievement of goals set out in *The NHS Long Term Plan*.^17^ Our findings suggest that work is still required to ensure that information from a wider variety of sources is easier to comprehend for much of the population in order for individuals to make informed decisions about health seeking and self-management of what can be a disabling condition for many. Health information providers should incorporate readability into their content design process to address the digital divide, incorporating shorter sentences and simpler words in their description of conditions and treatment.

It is important to note that readability is just one aspect in accessibility of information. Accessibility incorporates all disabilities that affect access to the web, including auditory, cognitive, neurological, physical, speech and visual^51^ and existing literature suggests that more attention is required in this hemisphere to improve access of online health information for people with disabilities.^52^ This is particularly important in the case of headache and migraine information as symptoms can be disabling; namely, increased sensitivity to light, poor concentration and visual problems.^53^ However, evidence on this subject is lacking and future research should also focus on different aspects of accessibility in order to improve universal access to health information.
